# Associations of media use and early childhood development: cross-sectional findings from the LIFE Child study

**DOI:** 10.1038/s41390-021-01433-6

**Published:** 2021-03-03

**Authors:** Clarissa Schwarzer, Nico Grafe, Andreas Hiemisch, Wieland Kiess, Tanja Poulain

**Affiliations:** 1 LIFE Leipzig Research Center for Civilization Diseases, Leipzig University, Leipzig, Germany; 2 Department of Women and Child Health, Hospital for Children and Adolescents and Center for Paediatric Research (CPL), Leipzig University, Leipzig, Germany

## Abstract

**Background:**

Excessive media usage affects children’s health. This study investigated associations between children’s and mother’s media use, parent–child interactions, and early-childhood development outcomes.

**Methods:**

Two hundred and ninety-six healthy 2–5-year-old preschoolers (52.4% male, mean age = 3.5 years) and 224 mothers from the LIFE Child cohort study were analyzed. Screen times and parent–child interactions were assessed using standardized parental questionnaires. Developmental skills were investigated using the standardized development test ET 6-6-R.

**Results:**

High screen times in children (>1 h/day) were significantly associated with lower percentile ranks in cognition (*b* = −10.96, *p* < 0.01), language (*b* = −12.88, *p* < 0.01), and social–emotional skills (*b* = −7.80, *p* = 0.05). High screen times in mothers (>5 h/day) were significantly associated with high media use by children (OR = 3.86, *p* < 0.01). Higher parent–child interaction scores were significantly associated with better body motor (*b* = 0.41, *p* = 0.05), cognition (*b* = 0.57, *p* < 0.01), language (*b* = 0.48, *p* = 0.02), and social–emotional outcomes (*b* = 0.80, *p* < 0.01) in children.

**Conclusions:**

Public health strategies should seek to educate caregivers as competent mediators for their children’s media habits, with focus on the need for children to have frequent parent–child interactions.

**Impact:**

- High media usage in children is related to poorer cognition, language, and social–emotional skills.
- More frequent parent–child interactions are associated with better body motor, cognition, language, and social–emotional skills in children.
- High level of media use in mothers is not directly related to children’s development outcomes but is directly related to high media usage of children.
- Public health strategies should seek to raise media awareness and management in both parents and children.

## Introduction

Early childhood is a period in which children reach crucial developmental milestones.^1^ Environmental factors may promote or hinder this sensitive process. Among these influencing factors, media exposure has been discussed with regard to its effect on early childhood development.^2,3^ World Health Organization (WHO) guidelines suggest limiting young children’s screen time to a maximum of 1 h per day.^4^ However, digital media have become ubiquitous in young children’s lives^5^ and, on average, preschoolers spend >2 h per day using digital media.^6^

Several previous studies have focused on the effects of excessive media exposure on children’s health.^7–10^ Longitudinal studies have indicated that greater levels of screen time are not only associated with poorer physical health and obesity in later life^7^ but also with lower psychological well-being and mental health issues in school-aged children and adolescents.^9,10^ Furthermore, previous longitudinal studies have demonstrated that high media use by preschool-aged children is related to conduct problems, hyperactivity, and inattention later in life.^11,12^ Importantly, longitudinal investigations have found high media exposure to be detrimental to early childhood development.^2,3,13^ In particular, young children’s cognition and language outcomes have been shown to be affected by high media consumption^3,13^ with toddlers and children from low socio-economic status (SES) families being especially vulnerable.^3,13,14^ Among the possible explanations put forward is that screens displace opportunities for children to communicate, interact, play, and therefore to learn.^2,3^ In contrast, other cross-sectional and longitudinal studies have found varied associations between preschooler’s and elementary-schooler’s media usage and development outcomes.^14–16^ Specifically, a stimulating environment,^14^ high-quality media content,^15^ and parental co-viewing^16^ have been identified as being beneficial for language and social–emotional outcomes in young children. In more recent literature, a meta-analysis has discussed touchscreen devices and interactive apps in terms of their potential for promoting early academic skills^17^ and a cross-sectional investigation found associations between fine motor abilities and early touchscreen scrolling in toddlers.^18^ In addition to the effects of children’s own media habits, previous cross-sectional and longitudinal studies have shown associations between parent’s media usage and behavioral problems in children <10 years of age.^19,20^ Parental media usage has been shown to disrupt parent–child interactions,^21^ with parents being slower, less attentive, and more passive in reacting to their children.^22^ Moreover, previous cross-sectional studies have found that parents’ conceptions play an important part in mediating preschool-aged children’s media habits^23,24^ and that parent–child interactions can moderate the relationship between high media exposure and young children’s executive functioning.^25^

Given the rapidly changing nature of digital media, for the study discussed here, we decided to investigate not only the use of television by German preschoolers and their mothers but also that of other forms of screen-based media (e.g., mobile phone/smartphone, personal computer (PC)/laptop/tablet, and game consoles). While former studies relied on screening tests based on parental reports^2^ or focused on single domains of childhood development,^3,13,14^ we used a detailed, standardized development test to evaluate associations between children’s media use and multiple dimensions of childhood development. Furthermore, as little is known about the relationships between mothers’ overall media use and their children’s development outcomes, we investigated these associations too. Finally, we included parent–child interactions in our analysis as we were interested in comparing the way children and mothers’ media behavior related to development outcomes to the way activities based on parent–child interaction related to such outcomes and whether these associations were independent from or potentially moderated by each other. Based on a number of previous longitudinal studies, we hypothesized negative associations between high overall screen time in children and early childhood development outcomes. Accordingly, we hypothesized that high media usage by mothers is negatively associated with early childhood development outcomes. In contrast, we hypothesized positive associations between the frequency of parent–child interactions and children’s development outcomes.

## Methods and measurements

### Subjects

Our research was conducted within the framework of the LIFE Child study, which is based at the Leipzig Research Center for Civilization Diseases at Leipzig University, Germany.^26,27^ LIFE Child is a cohort study that follows healthy children from the prenatal period to early adulthood and seeks to investigate environmental and lifestyle influences on young people’s health and development. All healthy subjects between the ages of 2 and 5 years who performed a specific development test were included in this analysis. Children born prematurely before the end of the 37th week of pregnancy (<37 + 0) were excluded. In cases of siblings, we included only one, randomly selected child from each family. For children with multiple visits, we included only one, randomly selected visit in our analysis. Data were collected between July 2017 and August 2019. The final sample included 296 children (52.4% boys, mean age = 3.5 years) and 224 mothers. SES was assessed using the Winkler index, whereby values assigned to parental education, parental occupation, and household equivalent income are combined to give an index score between 3 and 21 inclusive, with higher scores indicating higher SES.^28,29^ Using categories based on a representative German sample,^28^ 3% of our sample were placed in a low SES group (index score 3–8.4), 44% in a middle SES group (index score 8.5–15.4), and 53% in a high SES group (index score 15.5–21). Parents gave informed written consent before their children were included in the study. The study protocol was approved by the Ethics Committee of Leipzig University (Reg. No. 264-10-19042010), and the study was performed in accordance with the Declaration of Helsinki.

### Media use of children and mothers

The researchers assessed the children and mother’s media use using standardized parental questionnaires. The questionnaires were designed by the authors by adapting a previous questionnaire, which was lifted from the German Health Interview and Examination Survey for Children and Adolescents (KiGGS).^30^ The earlier version and its counterpart in the KiGGS study have been used in previous research,^10,11,31^ as has the adapted version used in our study.^20^ Parents were asked to report the time they and their children spend on average, on a daily basis, engaged with specific media devices. Leisure media usage was included, but media use at work and media not involving a screen (e.g., listening to music) were excluded. The questionnaires provided a range of possible answers from “not at all” to “>4 h per day”. The included devices were television/video/DVD (excluding computer screens), game console, computer/laptop/tablet with and without internet access, and mobile phone/smartphone with and without internet access.

Adapting the analysis method used in a previous study,^20^ the answers were converted into durations (hours/day) as follows: “not at all” = 0, “approximately 30 min/day” = 0.5, “approximately 1–2 h/day” = 1.5, “approximately 3–4 h/day” = 3.5, “>4 h/day” = 5. Media usage with and without internet access was added together. We added weekday and weekend usage using the formula ((usage on weekdays × 5) + (usage at weekends × 2))/7. Total screen time included durations of television/video/DVD, game console, computer/laptop/tablet, and mobile phone use. We categorized the total screen times reported for children as “normal” or “high” based on the WHO recommendation that young children should not exceed 1 h of screen time over the course of a day.^4^ We are not aware of any recommendations relating to total screen use by mothers. Therefore, the reported total screen times for mothers were categorized as “high” when they were greater than the average in the present sample. On this basis, a high reported total screen time for mothers was defined as >5 h/day. Hereafter, we will use the simplified terms of high screen time or high media usage when referring to high reported total screen time or high reported total media usage, respectively.

### Parent–child interactions

Interaction between parents and children was assessed using a questionnaire answered by parents that we adapted from the German version of the questionnaire on preschool-aged children’s activities in the family (Roßbach, H. G. & Leal, T. B., Mütterfragebogen zu kindlichen Aktivitäten im Kontext des Familiensettings (AKFRA). Deutsche Fassung des Questionnaire on Preschool-Aged Children’s Activities in the Family, 1993, unpublished manuscript). It covers 11 types of activity involving interaction between the child and all caregivers that belong to the child’s household: telling/reading a story, singing, movement-based play, drawing, building-based play, doing a jigsaw, ball play, role play, language play, number play, and talking about problems. The reported frequency for each activity was converted into a score, as follows: “once/month” = 1, “every 2 weeks” = 2, “once/week” = 3, “more often than once/week” = 4, “daily” = 5. The scores for each activity type were added to obtain an overall interaction score (max = 55). In our sample, Cronbach’s alpha was 0.76, indicating that the internal consistency of the questionnaire was satisfactory.

### Early childhood development

Early childhood development was assessed using the general development test “ET 6-6-R”, whose full German title translates as “Development Test 6 Months to 6 Years - Revision”.^32^ This standardized development test for infants, toddlers, and preschoolers aims to establish a differentiated developmental status and to detect developmental disorders or delays. The tasks used in the ET 6-6-R are based on “boundary stones,” i.e. skills targets that 90–95% of a normative, culturally similar population of children can be expected to have acquired by a certain age.^33^ The boundary stones themselves relate to established milestones of early childhood development. Body motor, hand motor, cognition, language, and social–emotional skills are evaluated, and the results converted into percentile ranks relative to a standardization sample. The ET 6-6-R comprises 79 parental questions and 166 tasks, with different requirements set for 13 age ranges, with the skills tested in older children building progressively on those tested in younger children. To ensure consistency in the test conditions, precise instructions are given on the setting, the task itself, and how it is to be evaluated.^34^ The ET 6-6-R has been also evaluated in terms of additional quality criteria. Specifically, in previous studies, it has been compared to the Bayley Scales of Infant Development BSID II, the SETK-2 language development test,^35^ and the WPSSI-IV intelligence test for preschoolers.^36^ These evaluations indicated significant intercorrelations within the ET 6-6-R as well as intercorrelations between the ET 6-6-R and the other tests, demonstrating a satisfactory level of internal and external validity. Moreover, language and cognition outcomes assessed by means of the ET 6-6-R were found to be reliable in predicting children’s intelligence quotients.^36^

### Data analysis

We performed our data analysis using R, version 3.5.3,^37^ using linear or logistic regression models to analyze associations between children’s media use, mothers’ media use, and parent–child interaction scores, and using linear regression models to investigate associations between these parameters and early childhood development outcomes. The percentile ranks in the single domains of development were included as outcome variables and children’s media use, mothers’ media use, and parent–child interaction scores were included as exposure variables. First, we calculated separate models for each exposure variable. Exposure variables that showed significant associations with at least one outcome variable were included together in the same model (multiple analyses) to test whether the associations were independent from one another. Furthermore, we investigated interactions between these exposure variables to examine whether the association between one pair of variables was moderated by another variable (moderator analysis).

All models were adjusted for the control variables of child age, child sex, and family SES and were evaluated for interactions with these control variables. We decided that interactions would be included in the final model if they satisfied certain preconditions with regard to significance (*p* < 0.05) and preservation of model quality (variance inflation factor <5). However, none of the interactions reached significance or limited the quality of the model by causing severe variance inflation. As such, the associations can be assumed not to differ depending on child age, sex, or SES. Strengths of association were described using non-standardized regression coefficients (*b*) or odds ratios (OR). The significance level was set to *α* = 0.05.

## Results

### Description of the study sample

Children’s media use, mothers’ media use, parent–child interaction scores, and the early childhood development percentiles are summarized in Table 1. The mean total screen time for the children was 0.75 h/day (SD = 0.70) with “high” screen times (≥1 h/day) reported for 24% of children. The mean total media use among mothers was 4.25 h/day (SD = 2.72) with “high” use of digital screens (>5 h/day) reported for 27% of mothers. The most common form of media use by children was watching television (0.55 h/day, SD = 0.56), whereas other digital devices were used rarely. The media device used most heavily by mothers was the mobile phone (1.63 h/day, SD = 1.40).

**Table 1:** Means (and standard deviations) of children’s media usage (h/day), mother’s media usage (h/day), parent–child interaction scores, and early childhood development percentile ranks in the present sample.

Measure	Possible range	Mean (SD)
Children’s media use (*n* = 296)		
TV	0–5	0.55 (0.56)
Game console	0–5	0.003 (0.02)
Mobile phone	0–10	0.08 (0.18)
PC/laptop/tablet	0–10	0.12 (0.28)
Total screen time^a^	0–30	0.75 (0.70)
% high screen time^b^		24%
Mothers’ media use (*n* = 224)		
TV	0–5	1.16 (1.10)
Game console	0–5	0.05 (0.21)
Mobile phone	0–10	1.63 (1.40)
PC/laptop/tablet	0–10	1.41 (1.40)
Total screen time^a^	0–30	4.25 (2.72)
% high screen time^c^		27%
Parent–child interactions (*n* = 296)		
Score	0–55	40.49 (7.17)
Early childhood development (*n* = 296)		
Body motor skills	0–100	38.52 (25.68)
Hand motor skills	0–100	44. 43 (27.21)
Cognition skills	0–100	45.50 (27.85)
Language skills	0–100	56.82 (28.00)
Social–emotional skills	0–100	57.16 (27.30)

^a^Total screen time: combination of TV, game console, mobile phone, PC/laptop/tablet.

^b^High screen time for children: >1 h/day.

^c^High screen time for mothers: >5 h/day.

The mean parent–child interaction score was 40.49 (SD = 7.17) indicating that each of the 11 activities involving both parents and children occurred on average more than once a week. The most common of these shared activities was telling/reading a story, which on average took place on a daily basis, followed by singing, moving-based play, building-based play, role play, and drawing, which on average took place more than once a week. Ball play, talking about problems, doing a jigsaw, and language play were reported as taking place once a week. The least frequent of the shared activities was number play, which on average took place once every 2 weeks.

Importantly, mothers with high media use were four times more likely than the other mothers to have children with high media use (OR = 3.86, 95% confidence interval (CI) 1.80 to 8.26, *p* < 0.01). In contrast, high screen times among mothers showed no significant association with the reported amount of parent–child interactions (*b* = −0.53, 95% CI −2.75 to 1.69, *p* = 0.64). Furthermore, high media use by children was not significantly associated with the amount of reported parent–child interactions (*b* = −1.60, 95% CI −3.65 to 0.45, *p* = 0.13).

### Associations between children’s media use and early childhood development outcomes

**Fig. 1:**
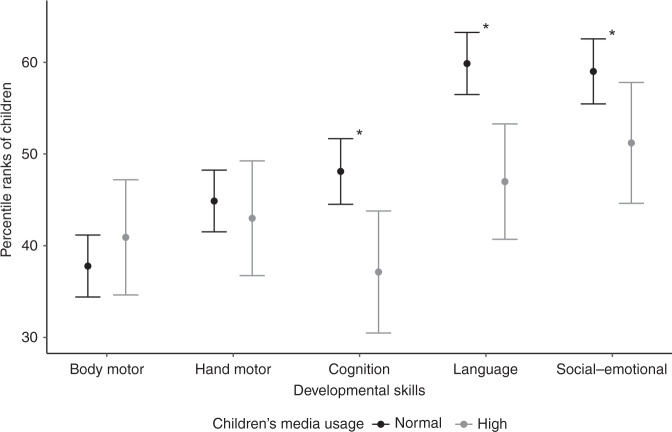
Effect plot (mean effect and 95% confidence interval) illustrating the estimated developmental percentile ranks of children by the level of media use (*n* = 296). For children, high media use was defined as >1 h/day and included TV, PC/laptop/tablet, mobile phone, and game console use. Asterisk (*) indicates the level of significance 0.05.

High screen times in children (>1 h/day) were significantly associated with lower percentile ranks in cognition (*b* = −10.96, 95% CI −18.69 to −3.24, *p* < 0.01), language (*b* = −12.88, 95% CI −20.19 to −5.57, *p* < 0.01), and social–emotional skills (*b* = −7.80, 95% CI −15.45 to −0.14, *p* = 0.05). In contrast, there were no significant associations between high media use by children and body motor (*p* = 0.40) or hand motor skills (*p* = 0.61). Figure 1 shows the children’s estimated percentile ranks by media use level (normal or high). By way of example, children with high media usage (>1 h/day) were estimated to score 10.96 points lower on the cognition scale and 12.88 points lower on the language scale than children with normal media usage.

Because high screen times in children (>1 h/day) were significantly associated with lower cognition, language, and social-emotional outcomes, we calculated separate models for usage of different types of media device. Since no specific guidelines exist for how long each media device should be used, we categorized reported TV usage times >0.5 h/day as high. For usage involving a games console, mobile phone, or PC/laptop/tablet, we compared users with non-users, i.e., usage >0 h/day was considered high. The distributions of normal versus high media usage times for the single media devices are presented in Supplementary Table S1. Supplementary Table S2 shows associations with the development outcomes cognition, language, and social–emotional skills. Most importantly, high TV screen time (>0.5 h/day) was significantly associated with lower cognition (*b* = −11.25, 95% CI −18.33 to −4.17, *p* < 0.01), and language outcomes (*b* = −7.28, 95% CI −14.09 to −0.47, *p* = 0.04), whereas high usage times (>0 h/day) involving a game console, mobile phone, or PC/tablet/laptop showed no significant associations with cognition, language, and social–emotional skills.

### Associations between mothers’ media use and early childhood development outcomes

**Fig. 2:**
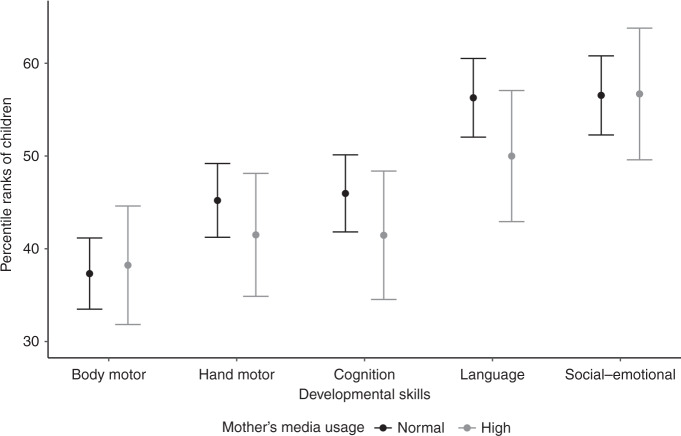
Effect plot (mean effect and 95% confidence interval) illustrating the estimated developmental percentile ranks of children by the level of mothers’ media use (*n* = 224). For mothers, high media use was defined as >5 h/day and included TV, PC/laptop/tablet, mobile phone, and game console usage.

High screen times among mothers (>5 h/day) showed no significant associations with percentile ranks in body motor (*p* = 0.81), hand motor (*p* = 0.35), cognition (*p* = 0.27), language (*p* = 0.14), or social–emotional skills (*p* = 0.97). Figure 2 displays the estimated percentile ranks of children by mother’s media use level (normal or high).

### Associations between parent–child interactions and early childhood development outcomes

Each additional point on the parent–child interaction score was associated with an increase of 0.41 percentile ranks on the body motor scale (95% CI 0.01–0.82, *p* = 0.05), an increase of 0.57 percentile ranks on the cognition scale (95% CI 0.14–1.00, *p* < 0.01), an increase of 0.48 percentile ranks on the language scale (95% CI 0.07–0.90, *p* = 0.02), and an increase of 0.80 percentile ranks on the social–emotional scale (95% CI 0.38–1.22, *p* < 0.01). For example, for children with a relatively low interaction score of 30 (indicating that each of the eleven activities involving parents and children occurred almost once a week), the estimated score on the cognition scale was 39.52 points, in contrast to children with a relatively high interaction score of 50 (indicating that each of the 11 activities involving parents and children occurred almost daily), who scored 50.94 points on the cognition scale. There were no significant associations between parent–child interaction scores and hand motor skills (*p* = 0.80). Associations between early childhood development outcomes and, variously, children’s media use, mothers’ media use, and parent–child interaction scores are summarized in Table 2.

**Table 2:** Associations of children’s media use (*n* = 296), mother’s media use (*n* = 224), and parent–child interaction scores (*n* = 296) with early childhood development percentile ranks^a^.

Dependent variables (*b*, 95% CI)^b^	Independent variables		
	High screen time of children^c^	High screen time of mothers^d^	Parent–child interaction score
Body motor skills	3.13 (−4.17 to 10.42)	0.90 (−6.60 to 8.40)	**0.41**^e^ **(0.01 to 0.82)**
Hand motor skills	−1.88 (−9.14 to 5.38)	−3.71 (−11.49 to 4.06)	0.05 (−0.35 to 0.46)
Cognition skills	−**10.96**^f^ **(**−**18.69 to** −**3.24)**	−4.52 (−12.64 to 3.60)	**0.57**^f^ **(0.14 to 1.00)**
Language skills	−**12.88**^f^ **(**−**20.19 to** −**5.57)**	−6.28 (−14.57 to 2.00)	**0.48**^e^ **(0.07 to 0.90)**
Social–emotional skills	−**7.80**^e^ **(**−**15.45 to** −**0.14)**	0.16 (−8.18 to 8.49)	**0.80**^f^ **(0.38 to 1.22)**

Bold values indicate significant associations.

^a^All associations are adjusted for age, sex, and SES.

^b^*b* = regression coefficient, non-standardized; 95% CI = 95% confidence interval.

^c^High screen time for children: >1 h/day.

^d^High screen time for mothers: >5 h/day.

^e^Level of significance 0.05.

^f^Level of significance <0.01.

### Associations between children’s media use, parent–child interaction scores, and early childhood development outcomes (multiple and moderator analysis)

As high media use by children and parent–child interaction scores were associated with child development in at least one domain, we included these parameters in multiple regression analyses (multiple analysis) and investigated whether associations between a particular parameter and developmental outcomes were moderated by another parameter (moderator analysis). In the multiple analysis, negative associations between high screen time in children and their development outcomes remained significant for cognition skills (*b* = −10.13, 95% CI −17.83 to −2.44, *p* = 0.01), and language skills (*b* = −12.20, 95% CI −19.50 to −4.90, *p* < 0.01). The negative associations between high screen time in children and their social–emotional skills did not remain significant (*b* = −6.57, 95% CI −14.10 to 0.97, *p* = 0.09) after controlling for parent–child interactions. The positive associations between parent–child interactions and early childhood development outcomes remained significant for body motor skills (*b* = 0.43, 95% CI 0.03 to 0.84, *p* = 0.04), cognition skills (*b* = 0.52, 95% CI 0.09 to 0.95, *p* = 0.02), language skills (*b* = 0.42, 95% CI 0.02 to 0.83, *p* = 0.04), and social–emotional skills (0.77, 95% CI 0.35 to 1.19, *p* < 0.01) after controlling for high media use by children. The moderator analysis revealed no significant interactions between children’s media use and parent–child interactions in relation to body motor skills (*b* = −0.03, 95% CI −0.93 to 0.88, *p* = 0.96), cognition skills (*b* = −0.13, 95% CI −1.09 to 0.82, *p* = 0.78), language skills (*b* = −0.61, 95% CI −1.52 to 0.30, *p* = 0.19), and social–emotional skills (*b* = −0.43, 95% CI −1.37 to 0.51, *p* = 0.37).

## Discussion

### Principal findings

In our present study, German preschoolers on average spent approximately 45 min each day engaged with digital media. As such, the average screen time was within the WHO’s recommended limit for preschoolers of a maximum of 1 h/day media exposure.^4^ However, 24% of children exceeded the recommended level. In line with our hypothesis, high media use by children was related to poorer cognition, language, and social–emotional development outcomes. A more detailed analyses revealed that especially high TV usage in children was associated with poorer cognitive and language development outcomes. In contrast, high media use by mothers (>5 h/day) was not directly related to early childhood development outcomes but was significantly associated with high media use in children. Increasing parent–child interactions were associated with better body motor, cognition, language, and social–emotional outcomes in children.

### Associations of media use, parent–child interactions, and early childhood development

Consistent with previous studies, our findings suggest adverse effects of high media exposure on cognition, language, and social–emotional skills in children.^2,3,13^ Possible reasons for this are that high media usage displaces children’s opportunities to communicate, interact, and play and that related conduct problems, such as hyperactivity and inattention,^11,12^ reduce children’s ability to learn. Looking at the usage of different media devices, we can attribute negative associations between high media usage in children and their cognition and language outcomes primarily to high TV screen time, there being little use of other media devices. In 2–5-year-old children, TV is still the most used medium. The fact that we did not find associations between the use of other media devices and development outcomes might be explained by the infrequent use (rather than insignificance) of these media devices. Moreover, multiple and moderator analyses revealed that negative associations between high media usage in children and cognition and language skills were independent from and did not differ depending on the frequency of parent–child interactions. Unlike the findings of prior studies,^25^ these results suggest that parent–child interactions may not serve to moderate the effects of high media usage with respect to children’s developmental outcomes. In contrast, our findings do support the idea that screen-based media usage offers suboptimal stimulation for cognitive and linguistic development,^38^ with this appearing to be independent of the effects of stimulating forms of parent–child interaction. In our sample, body and hand motor skills were not affected by high screen times in children. Possible reasons are that children remain physically active despite their high media usage and increasingly use mobile devices with interactive apps that can be carried around easily.^18^ Overall, our findings indicate that exposing children to >1 h of digital media interferes with sensitive development processes during infancy.

Previous studies have suggested that parental distraction by media devices could have detrimental effects on parent–child interaction.^19–22^ In our analysis, high media use in mothers was not associated with less frequent parent–child interactions nor with poorer development outcomes in children. However, the questionnaire used did not assess the media use of mothers in the presence of the child or during shared activities. As they stand, these findings imply that media use by mothers does not displace shared activities with their children and does not directly affect development outcomes in children. Nevertheless, mothers who exhibited high levels of daily digital media use were more likely to have children with high screen times. Therefore, mothers should be aware that, in addition to their perception of media use,^22,23^ their personal media habits operate as mediators for their children’s media use. Finally, we observed positive associations between parent–child interaction scores and body motor, cognition, language, and social–emotional outcomes in the children. These were independent from the children’s level of media use. This implies that shared activities involving parents and children stimulate and protect sensitive development processes in children.

### Strengths and weaknesses

The strengths of our study include the fact that it offers an up-to-date assessment of young children’s media use, mothers’ media use, and parent–child interactions. Furthermore, the ET 6-6-R belongs to the newest generation of development tests for German speaking countries and addresses language and cognition outcomes, which are reliable predictors for children’s intelligence quotients.^36^

The limitations include the fact that it relies on reports of media usage based on maternal perceptions and is thus susceptible to self-presentation biases. Furthermore, the type of media content and the context in which media devices were used (in co-use with a caregiver or alone) were not evaluated. The categories of “high” and “normal” media exposure in mothers were based on observations in this study, since we are not aware of any available guidelines on media consumption for mothers. Moreover, the parent–child interaction questionnaire did not assess durations or quality of interactions and identify the person(s) the activity was shared with (mother or father). Finally, the sample was shifted to higher levels of SES and is not representative of German families.

### Implications and future research

Given the high number of children exceeding the recommended limits of 1 h of media use per day and the adverse effects of high media consumption on children’s development, the scope of educational campaigns and public health strategies should include the question of media awareness and management in both parents and children. In doing so, policymakers, health care professionals, and educators need to consider strategies for encouraging appropriate, guided media use with adequate safeguards in family and preschool-education settings. Therefore, the focus should not only be on implementing rigid daily limits on screen time but also on the communal, interactive use of high-quality media content.^14,16^ Most importantly, education is needed for caregivers, in order that they become competent mediators for their children’s media habits, with sufficient focus on the need for children to have frequent parent–child interactions.

Further investigations should examine the long-term effects of digital media on childhood development, with a focus on protective factors such as parental mediation and co-use and the potential impacts of interactive touchscreen devices in young children.

## Conclusions

Our findings suggest that >1 h of daily media usage in preschoolers is associated with poorer cognition, language, and social–emotional skills, whereas parent–child interactions are related to better body motor, cognition, language, and social–emotional skills in children. Moreover, our study indicates that high levels of media use in mothers is not directly related to children’s development outcomes but is directly related to high media usage of children.

With regards to the international trend that has seen young children become increasingly involved with the digital world, caregivers need to be sensitized and educated as mediators and role models for their children’s media habits. As part of this process, caregivers will be required to moderate their own media habits, with a view to offering their children more in the way of parent–child interactions.

## Supplementary information

Supplementary Information

## References

[1] Michaelis R, Niemann G (2004). Entwicklungsneurologie und Neuropädiatrie [Developmental Neurology and Neuropediatrics].

[2] Madigan S, Browne D, Racine N, Mori C, Tough S (2019). Association between screen time and children’s performance on a developmental screening test. JAMA Pediatr..

[3] Tomopoulos S (2010). Infant media exposure and toddler development. Arch. Pediatr. Adolesc. Med..

[4] World Health Organisation. Guidelines on physical activity, sedentary behaviour and sleep for children under 5 years of age. https://apps.who.int/iris/bitstream/handle/10665/325147/WHO-NMH-PND-2019.4-eng.pdf (2019). 31091057

[5] Ofcom. Children and parents: media use and attitudes report 2019. https://www.ofcom.org.uk/research-and-data/media-literacy-research/childrens/children-and-parents-media-use-and-attitudes-report-2019 (2020).

[6] Common Sense Media. The common sense census: media use by kids age zero to eight. https://www.commonsensemedia.org/research/the-common-sense-census-media-use-by-kids-age-zero-to-eight-2017 (2017).

[7] Hancox RJ, Milne BJ, Poulton R (2004). Association between child and adolescent television viewing and adult health: a longitudinal birth cohort study. Lancet.

[8] Dennison BA, Erb TA, Jenkins PL (2002). Television viewing and television in bedroom associated with overweight risk among low-income preschool children. Pediatrics.

[9] Twenge JM, Campbell WK (2018). Associations between screen time and lower psychological well-being among children and adolescents: evidence from a population-based study. Prev. Med. Rep..

[10] Poulain T (2019). Reciprocal longitudinal associations between adolescents’ media consumption and psychological health. Acad. Pediatr..

[11] Poulain T (2018). Reciprocal associations between electronic media use and behavioral difficulties in preschoolers. Int. J. Environ. Res. Public Health.

[12] Christakis DA, Zimmerman FJ, DiGiuseppe DL, McCarty CA (2004). Early television exposure and subsequent attentional problems in children. Pediatrics.

[13] Zimmerman FJ, Christakis DA (2005). Children’s television viewing and cognitive outcomes: a longitudinal analysis of national data. Arch. Pediatr. Adolesc. Med..

[14] Bittman M, Rutherford L, Brown J, Unsworth L (2011). Digital natives? New and old media and children’s outcomes. Aust. J. Educ..

[15] Rice ML, Huston AC, Truglio R, Wright J (1990). Words from ‘Sesame Street’: learning vocabulary while viewing. Dev. Psychol..

[16] Rasmussen EE (2016). Relation between active mediation, exposure to Daniel Tiger’s Neighborhood, and US preschoolers’ social and emotional development. J. Child. Media.

[17] Griffith SF, Hagan MB, Heymann P, Heflin BH, Bagner DM (2020). Apps as learning tools: a systematic review. Pediatrics.

[18] Bedford R, Saez de Urabain IR, Cheung CHM, Karmiloff-Smith A, Smith TJ (2016). Toddlers’ fine motor milestone achievement is associated with early touchscreen scrolling. Front. Psychol..

[19] McDaniel BT, Radesky JS (2018). Technoference: longitudinal associations between parent technology use, parenting stress, and child behavior problems. Pediatr. Res..

[20] Poulain T, Ludwig J, Hiemisch A, Hilbert A, Kiess W (2019). Media use of mothers, media use of children, and parent–child interaction are related to behavioral difficulties and strengths of children. Int. J. Environ. Res. Public Health.

[21] Radesky J (2015). Maternal mobile device use during a structured parent-child interaction task. Acad. Pediatr..

[22] Kirkorian HL, Pempek TA, Murphy LA, Schmidt ME, Anderson DR (2009). The impact of background television on parent–child interaction. Child. Dev..

[23] Nikken P, Schols M (2015). How and why parents guide the media use of young children. J. Child Fam. Stud..

[24] Hinkley T, Carson V, Kalomakaefu K, Brown H (2017). What mums think matters: a mediating model of maternal perceptions of the impact of screen time on preschoolers’ actual screen time. Prev. Med. Rep..

[25] Linebarger DL, Barr R, Lapierre MA, Piotrowski JT (2014). Associations between parenting, media use, cumulative risk, and children’s executive functioning. J. Dev. Behav. Pediatr..

[26] Quante M (2012). The LIFE child study: a life course approach to disease and health. BMC Public Health.

[27] Poulain T (2017). The LIFE child study: a population-based perinatal and pediatric cohort in Germany. Eur. J. Epidemiol..

[28] Lampert PDT, Müters S, Stolzenberg H, Kroll LE (2014). Messung des sozioökonomischen Status in der KiGGS-Studie [Measuring the socio-economic status in the KiGGS-study].. Bundesgesundheitsbl.

[29] Winkler, J. & Stolzenberg, H. Adjustierung des Sozialen-Schicht-Index für die Anwendung im Kinder-und Jugendgesundheitssurvey (KiGGS) [Adjusting the social-stratum-index for the application in the children- and adolescents-survey (KiGGS)]. Wismarer Diskussionspapiere 07. http://hdl.handle.net/10419/3919 (2009).

[30] Hölling H (2012). The KiGGS study. Nationwide representative longitudinal and cross-sectional study on the health of children and adolescents within the framework of health monitoring at the Robert Koch Institute. Bundesgesundheitsbl.

[31] Manz K (2014). Physical activity and electronic media use in children and adolescents: results of the KiGGS study: first follow-up (KiGGS wave 1). Bundesgesundheitsbl.

[32] Macha T, Petermann F (2008). The ET 6–6: a method for developmental assessment for German-speaking countries. J. Psychol..

[33] Michaelis R, Berger R, Nennstiel-Ratzel U, Krägeloh-Mann I (2013). Validierte und teilvalidierte Grenzsteine der Entwicklung: ein Entwicklungsscreening für die ersten 6 Lebensjahre [Validated and semi-validated boundary stones of development: development screening in the first six years of life]. *Monatsschr. Kinderheilkd.*. Monatsschr. Kinderheilkd..

[34] Macha T, Petermann F (2013). Objektivität von Entwicklungstests: zur Standardisierung der entwicklungsdiagnostischen Befunderhebung [Objectivity of development tests: about the standardization of development diagnostic surveys]. Diagnostica.

[35] Lissmann I, Domsch H, Lohaus A (2006). Zur Stabilität und Validität von Entwicklungstestergebnissen im Alter von sechs Monaten bis zwei Jahren: eine Analyse am Beispiel des ET 6-6 [About the stability and validity of development test findings in children at the age of six months to two years: an analysis by the example of the ET 6-6]. Kindh. Entwickl..

[36] Walter F, Petermann F, Daseking M (2018). Vorhersage von kognitiven Fähigkeiten in der WPSSI-IV durch den ET 6-6-R [Prediction of cognitive skills in the WPSSI-IV using the ET 6-6-R]. Kindh. Entwickl..

[37] R Core Team. *R: A Language and Environment for Statistical Computing* (R Foundation for Statistical Computing, 2016).

[38] Hutton JS, Dudley J, Horowitz-Kraus T, DeWitt T, Holland SK (2020). Associations between screen-based media use and brain white matter integrity in preschool-aged children. JAMA Pediatr..

