# Supplementary material for: Associations of media use and early childhood development: cross-sectional findings from the LIFE Child study
Source: Pediatr Res. 2021 Mar 3;91(1):247–53. doi: 10.1038/s41390-021-01433-6 (PMC8770129; doi:10.1038/s41390-021-01433-6)
Supplement: Supplementary file 1 — Supplementary Information [file 41390_2021_1433_MOESM1_ESM.docx]

**SUPPLEMENTS**

| **Media device** | **Children’s high media use (n, %)** |
| --- | --- |
| TV ^a^ | 100 (34 %) |
| Game console ^b^ | 7 (2 %) |
| Mobile phone ^b^ | 64 (22 %) |
| Pc/laptop/tablet ^b^ | 79 (27 %) |
| Total screen time ^c^ | 70 (24 %) |

Table 1: Media devices and numbers of children showing high media usage (n = 296)

^a^ High TV screen time: > 0.5 h/day; ^b^ High game console, mobile phone and pc/laptop/tablet screen time: > 0.0 h/day; ^c^ Total screen time: combination of TV, game console, mobile phone, Pc/laptop/tablet, high screen time: > 1 h/day

Table 2: Associations of children’s media device use (n = 296) with early childhood development percentile ranks ^a^

| **Dependent**  **variables**  **(b, 95 % CI)^b^** | **Independent variables** | | | |
| --- | --- | --- | --- | --- |
|  | High TV screen time ^c^ | High game console usage ^d^ | High mobile phone usage ^d^ | High pc/ laptop/tablet  usage ^d^ |
| Cognition  skills | **-11.25 ****  **(-18.33 to -4.17)** | -9.18  (-29.81 to 11.45) | -1.80  (-9.42 to 5.83) | -5.34  (-12.72 to 2.04) |
| Language  skills | **-7.28 ***  **(-14.09 to -0.47)** | 6.23  (-13.44 to 25.90) | -3.29  (-10.55 to 3.97) | -5.48  (-12.51 to 1.55) |
| Social-emotional skills | -4.89  (-11.95 to 2.18) | -14.97  (-35.23 to 5.29) | -2.84  (-10.35 to 4.66) | -3.22  (-10.50 to 4.06) |

^a^ All associations are adjusted for age, sex and SES; ^b^ b = regression coefficient, non-standardized, 95 % CI = 95 % confidence interval; ^c^ High TV screen time: > 0.5 h/day; ^d^ High game console, mobile phone and pc/laptop/tablet screen time: > 0 h/day; * level of significance 0.05; ** level of significance < 0.01.
